# Specialist technician-entomologists adjustment for malaria control in endemic setting of Iran

**DOI:** 10.1186/1475-2875-11-S1-P111

**Published:** 2012-10-15

**Authors:** Hamid Reza Basser, Khandan Shahande, Davood Shojaeizadeh, Esmaeil Shojaeizadeh

**Affiliations:** 1 Department of Medical Entomology & Vector Control, School of Public Health, Tehran University of Medical Sciences, Tehran, lran; 2 Department of Health Education and Promotion, School of Public Health, Tehran University of Medical Sciences, Tehran, lran; 3 Center for Community Based Participatory Research. Tehran University of Medical, Iran; 4 Department of Health Education and Promotion, School of Public Health, Tehran University of Medical Sciences, Tehran, Iran; 5

## Background

Effective malaria control programmes prevent malaria transmission by promoting personal protection measures and effective vector control strategies, and providing appropriate case management with early diagnosis and effective treatment. The objective of the study was to understanding of malaria entomologist in relation to current malaria control in their area of activities and technical disciplines.

## Methods

A qualitative approach was adopted based on a semi structured interview with 30 individuals working at community-level in malaria endemic areas. Thematic content analysis was used to analysis the data.

## Results

Although most of participants have been concern with the distribution and causation of problems of malaria control, few of them have addressed the issue of solutions. Participants had positive perceptions on their basic activities and opinions in relation to biologic and epidemiologic factors in their field work. In contrary, their perceptions in relation to malaria control policy and integrated management of vector control were rather negative.

## Conclusion

There was not feedback mechanism on malaria control activity among practitioners. Therefore, any problems due to the mismatch between the institutional tasks and individual role performance should be fed back into the health system and adjustment should be made. Greater emphasizes should be given to the choice of solutions.
